# Application of Extreme Learning Machine in the Survival Analysis of Chronic Heart Failure Patients With High Percentage of Censored Survival Time

**DOI:** 10.3389/fcvm.2021.726516

**Published:** 2021-10-29

**Authors:** Hong Yang, Jing Tian, Bingxia Meng, Ke Wang, Chu Zheng, Yanling Liu, Jingjing Yan, Qinghua Han, Yanbo Zhang

**Affiliations:** ^1^Department of Health Statistics, School of Public Health, Shanxi Medical University, Taiyuan, China; ^2^Shanxi Provincial Key Laboratory of Major Diseases Risk Assessment, Taiyuan, China; ^3^Department of Cardiology, The First Hospital of Shanxi Medical University, Taiyuan, China

**Keywords:** chronic heart failure, survival analysis, extreme learning machine, random survival forest, clinical prediction model

## Abstract

**Objective:** To explore the application of the Cox model based on extreme learning machine in the survival analysis of patients with chronic heart failure.

**Methods:** The medical records of 5,279 inpatients diagnosed with chronic heart failure in two grade 3 and first-class hospitals in Taiyuan from 2014 to 2019 were collected; with death as the outcome and after the feature selection, the Lasso Cox, random survival forest (RSF), and the Cox model based on extreme learning machine (ELM Cox) were constructed for survival analysis and prediction; the prediction performance of the three models was explored based on simulated data with three censoring ratios of 25, 50, and 75%.

**Results:** Simulation results showed that the prediction performance of the three models decreased with increasing censoring proportion, and the ELM Cox model performed best overall; the ELM Cox model constructed with 21 highly influential survival predictors screened from actual chronic heart failure data showed the best performance with C-index and Integrated Brier Score (IBS) of 0.775(0.755, 0.802) and 0.166(0.150, 0.182), respectively.

**Conclusion:** The ELM Cox model showed good discrimination performance in the survival analysis of patients with chronic heart failure; it performs consistently for data with a high proportion of censored survival time; therefore, the model could help physicians identify patients at high risk of poor prognosis and target therapeutic measures to patients as early as possible.

## Introduction

Chronic heart failure (CHF), one of the most severe cardiovascular diseases of the 21st century (1), is a complex clinical syndrome manifested when the heart does not pump enough blood for tissue and metabolic needs (2). As the prevalence of heart failure in China increases year by year, it has become a major cause of hospitalization and rehospitalization among the elderly, imposing a heavy medical burden on individuals and society (3). Adverse prognosis in heart failure patients can be intervened promptly with lifestyle modifications and medications that effectively slow the progression of the disease or prevent the onset of adverse prognosis (4).

Therefore, a prediction model for people with HF is beneficial to the development of patients, doctors, and even the entire society. Doctors can prescribe more aggressive treatment plans for high-risk patients based on accurate risk prediction, and patients will follow the treatment more because they have confidence in the treatment plan prescribed by the doctor (5). An accurate prediction model can also help clinical researchers design clinical trials to target high-risk patients with heterogeneous characteristics and change treatment interventions (6). Multiple heart failure survival prediction models have been developed and verified in multiple cohorts, such as the Seattle heart failure prediction model (7, 8), and the above prediction models have been successfully used in routine clinical care to manage patients with different degrees of heart failure. However, the above survival prediction model data comes from clinical trials. These data have a small sample size, strict test conditions, lack of heterogeneity in the patient population, and poor population representation (9). In addition, these models based on clinical trials are not derived from real-world data. Even if such a model is constructed with high accuracy, it is not very useful for real-world research (10). As electronic medical records (EHRs) become more common in clinical research, methods for predicting the prognosis of HF using EHRs instead of clinical trial data have become necessary (11, 12).

In recent years, with the rapid development of artificial intelligence, machine learning technology has been used to build cardiovascular disease prediction models more and more widely (13–15). In models for aging patients, many studies have also proved that the prediction performance of the survival model based on machine learning is better than the traditional Cox proportional hazard model (16). Survival analysis models the time to event (17). A major challenge in survival analysis is censoring, which is the problem that makes the modeling time of event data more complicated, compared with traditional regression methods (18–21). Miao (22) used the Cox and RSF models to predict cardiovascular disease in 2015 and assessed the performance of the constructed models by comparing the discrimination ability, the identification of nonlinear effects, and the identification of significant predictors, and the results showed that the RSF model could automatically identify nonlinear effects among variables, while the Cox model could not. However, the RSF model was not as good as the Cox model in identifying some variables with small population proportional distribution. Therefore, the Cox model cannot be completely replaced by the RSF model in survival analysis.

Hong (23) applies the emerging extreme learning machine (ELM) algorithm to the survival analysis of a single-layer feedforward neural network. It performs well in high-dimensional and ultra-high-dimensional real data sets. The results show that ELM Cox has good predictive performance. In addition, it also has a greater advantage in shortening the calculation time (24). Wang (25) proposed an ELM survival model in 2018 that could effectively solve the above problems. Wang (26) applied the ELM algorithm to survival analysis and showed the ELM Cox model's good prediction performance on high and ultra-high dimensional datasets and reduced computation time.

In this study, we used the EHRs of inpatients with heart failure to construct least absolute shrinkage and selection operator Cox regression model (Lasso Cox), RSF, and ELM Cox survival analysis prognostic models. According to VIMP and minimal depth method, the predictors that have a significant impact on the prognosis are selected out, and a model with high predictive ability is constructed. To provide the basis for patients, doctors, and clinical researchers to initiate subsequent treatment and intervention measures.

## Objects and Methods

### Sources of Information

Data in this study are from the complete inpatient medical records of patients diagnosed with CHF in the cardiology departments of two grade 3 and first-class hospitals in Taiyuan, Shanxi Province during the period Jan. 2014 to Apr. 2019. The data were obtained according to the case report form of chronic heart failure (CHF-CRF) developed by our research group according to the case record content and HF guidelines (27). Patients were followed up at 3, 6, and 12 months after discharge and every 6 months after that until July 2019. The primary outcome is CHF-related mortality. Inclusion criteria are patients aged ≥18 years presenting with typical signs or symptoms of CHD, in NYHA class II to IV, and receiving heart failure medications or other therapeutic measures. Patients were excluded if they had experienced an acute cardiovascular event within the past 2 months, they had a psychiatric disorder or other major non-cardiovascular chronic disease.

### Statistical Analysis

SPSS (V26.0) and R 3.6.5 were used for statistical analysis. For group comparisons, we used chi-square tests for categorical variables; Student's *t*-test or nonparametric Kruskal-Wallis tests for continuous variables. Univariate Cox regression analysis was used to describe the influence of variables on primary outcomes. Random forest VIMP (variable Importance) and minimal depth (28) methods are used to select variables. Significance thresholdα = 0.05. The R packages *SurvELM* (29), *randomForestSRC* (30), and *glmnet* (31) are used to build the ELM Cox, RSF, and Lasso Cox survival models.

### Data Preprocessing and Feature Selection

**Figure 1:**
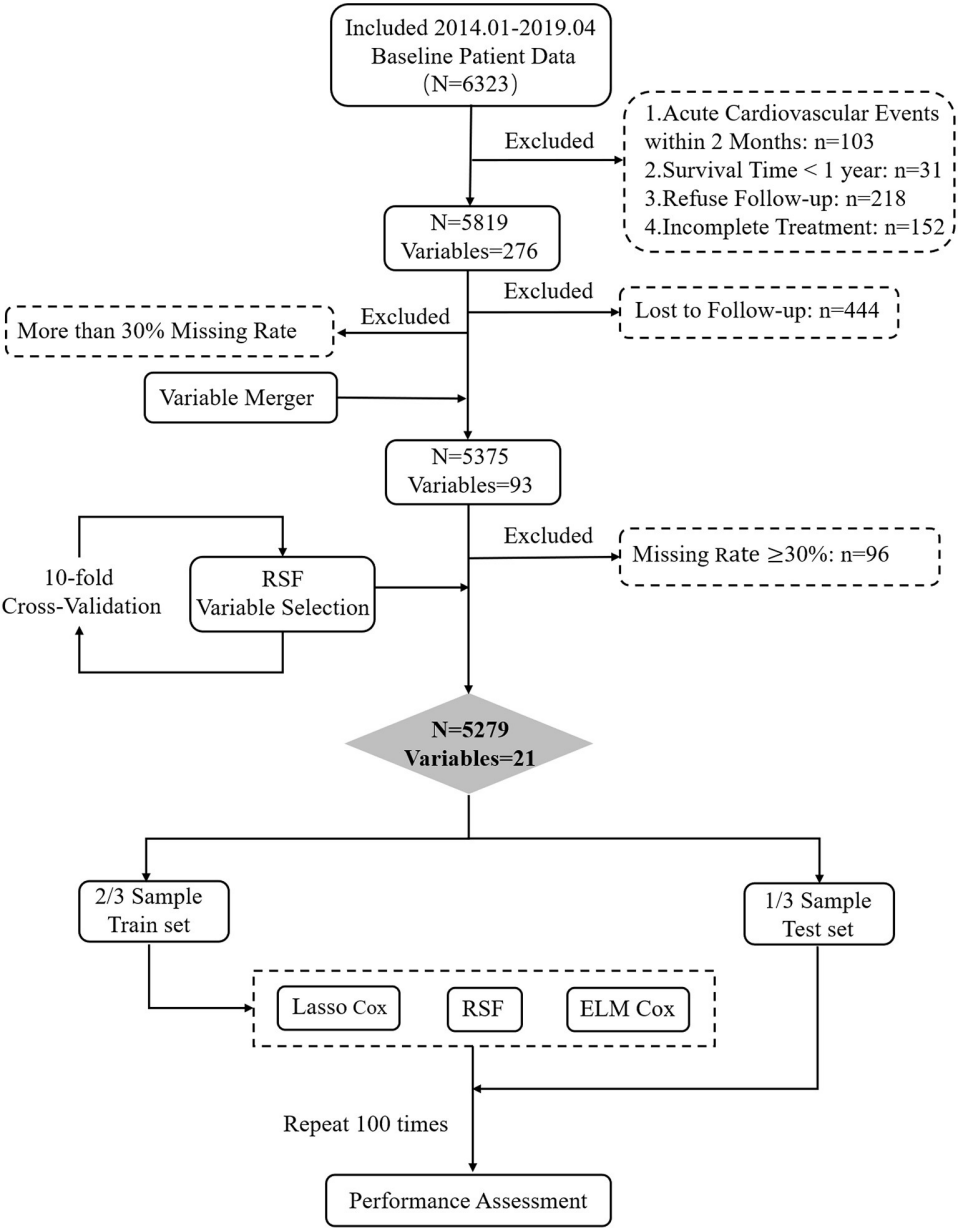
A flowchart describing the general framework of the study.

In clinical practice, patients undergo different tests, resulting in missing indicators in the data collected. Variables with ≥30% missing were removed from the analysis (Supplementary Table 3). According to previous research (32), this paper uses the MissForest algorithm in the *missForest* R package (33) to impute variables with <30% missing rate. We use random forest's VIMP and minimal depth method to carry out 5-fold cross-validation to select variables for constructing predictive models. The research process is shown in Figure 1 (Details in Supplementary Materials).

## Research Methodology

### The Lasso Cox Model

Lasso is a regression analysis method that performs regularization along with variable selection to improve the prediction performance and interpretability of statistical models. Tibshirani (34) applied Lasso to the Cox proportional hazards model in 1997 and performed variable selection by reducing the absolute values of the penalty coefficients to even zero so that the estimated variance of the final model was decreased and its interpretability increased.

### Random Survival Forest

RSF is an algorithm that estimates risks under the framework of the random forests using statistical methods without making any assumptions about individual risk functions. RSF randomly selects the features and samples of subtrees and uses the log-rank test to split the trees; the overall cumulative risk function is estimated after calculating the cumulative risk function for each tree. RSF extends the application of Breiman's Random Forests method for truncated data with advantages such as being free from the assumption of equal scaling conditions and suitability for complex variable problems with variable multicollinearity and high dimensionality (35).

### The Cox Model Based on Extreme Learning Machine

Some recent interesting studies have shown that when the assumptions of classic parametric or semi-parametric survival models [such as the Cox (1972) model] are seriously violated, neural network models are useful alternatives in modeling survival data (23). The Faraggi-Simon method is a feedforward neural network nonlinear proportional hazard model. This method uses the nonlinear output function of the neural network to replace the linear combination of covariates and optimizes the improved Cox partial likelihood estimation coefficient. Therefore, the Faraggi-Simon method (36) is generally regarded as a nonlinear extension of the Cox model and a classic proportional hazard model with the most advantages (23, 37). Wang (29) introduced the ELM algorithm into survival analysis and proposed a new regularized Cox model based on the simple framework of the Faraggi-Simon method.

There are several reasons why we choose ELM as the single-hidden-layer feedforward neural network (SLFN) Cox model instead of other popular deep neural network survival models. First, it has been proved that any continuous objective function can be approximated by SLFN with adjustable hidden nodes. This means that complex network structures such as MLP neural networks or deep neural networks may not always be necessary (38, 39). Second, most of the backpropagation or similar algorithms used in deep learning neural networks adjust the input and output weights and hidden layer bias values through optimization based on gradient descent. This is likely to reduce the generalization ability of the network. In contrast, ELM hidden node parameters do not need to be adjusted, and better model performance can be obtained without complicated parameter tuning (40). Third, the simulation study of Wang et al. (23) showed that ELM Cox can choose a simple linear kernel in various types of data, and has good stability under different ratios of censoring conditions. This may be the linear check is not sensitive to Kernel parameter *c* (41).

### Model Development

Censoring can have an important influence on the results of survival analysis. A high degree of censoring can result in lower accuracy and effectiveness of a model, increasing the risk of bias (42). The censored rate of heart failure data in this study was 90.2%. To build a stable performance model, we used stratified bootstrap (43). In this study, we stratified the training sets and the testing sets in the ratio of 2:1 by the outcome. To obtain reliable model indicators, the entire process was repeated 100 times, and the performance of the model was compared.

The parameter combination of the RSF model with the optimal prediction performance was selected through 5-fold cross-validation, i.e., ntree = 500, mtry = 7, and nodesize = 60; ELM Cox model was constructed with the default parameters, i.e., implied layer nodes L = 100 and regularization parameter C = 1e5.

### Model Evaluation Metrics

Two common survival analysis evaluation metrics, Integrated Brier Score (IBS) (44) and Harrell's concordance index (C-index) (20) were used to assess the accuracy of the survival analysis models in the follow-up experiments. The C-index for survival prediction indicates the proportion of observations with correct ranking divided by all valid pairs, and the closer C-index is to one, the better the model prediction; IBS is the Brier score of the survival model over a certain period, and the smaller the IBS, the stronger the prediction model. Comparisons of indicators between models were made using nonparametric rank-sum tests and Nemenyi *post hoc* tests.

### Simulation Analysis

**Figure 2:**
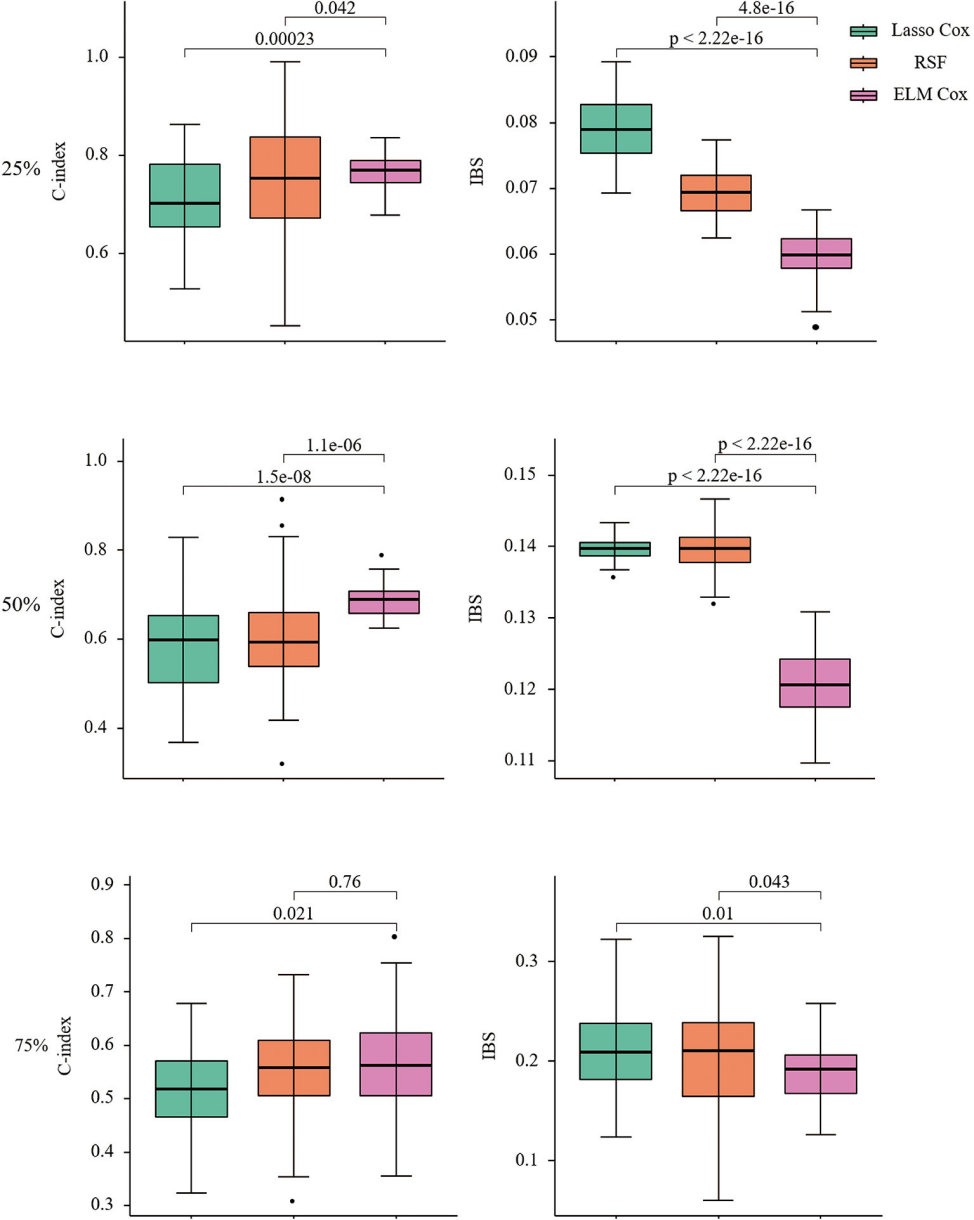
C-index and IBS of Lasso COX/RSF/ELM Cox model at different censoring ratios. Nonparametric Friedman test and Nemenyi *post hoc* test were used to make comparison with the ELM Cox group, *P* < 0.05 means statistically significant.

In this paper, the R package *SimSurv* (45) was used to test the applicability of the Lasso Cox, RSF, and ELM Cox algorithms to low-dimensional data, in which the fundamental risk function was set to be Weibull distributed and the scale parameter was set to two to give a simulation dataset with 1,000 samples and five normal covariates (23). We generated on the data set and were still alive until the end of follow-up, that is, the proportion of censoring was 25, 50, and 75%. And the three models were constructed by repeating 50 times with default parameters. The results are shown in Figure 2.

When the censoring ratio is 25%, the performance of RSF and ELM Cox models is almost the same with a C-index >0.75. The evaluation indexes of the two models have a small fluctuation range, indicating relatively good performance. The Lasso Cox model performed slightly worse, but the results were still acceptable. The IBS of the three models is all below 0.1, indicating that their overall performance is stable. The ELM Cox model outperformed the other two models when the censoring ratio was 50%. At a censoring ratio of 75%, the performance of all three models decreased, with a C-index below 0.6 and IBS over 0.15. In summary, the performance of the three prediction models gradually decreases as the survival time data censoring ratio increases and the ELM Cox model performs most consistently among the three constructed models. Performance comparison of the three algorithms in low-dimensional data shows that the ELM Cox model can be applied in the survival analysis of heart failure patients.

## Results

### Basic Information

According to the inclusion and exclusion criteria, at the end of follow-up, a total of 5,819 patients were included in the study, of which 444 (7.63%) were excluded due to loss to follow-up. Five thousand two hundred seventy-ninth patients were finally enrolled, of which 4,762 (90.2%) were alive and 517 (9.8%) died. The mean age of the enrolled patients was (70 ± 11.7) years, with 3,404 (64.5%) male and 1,875 (35.5%) female cases (Details in Supplementary Table 1).

### Univariate Cox Regression

**Figure 3:**
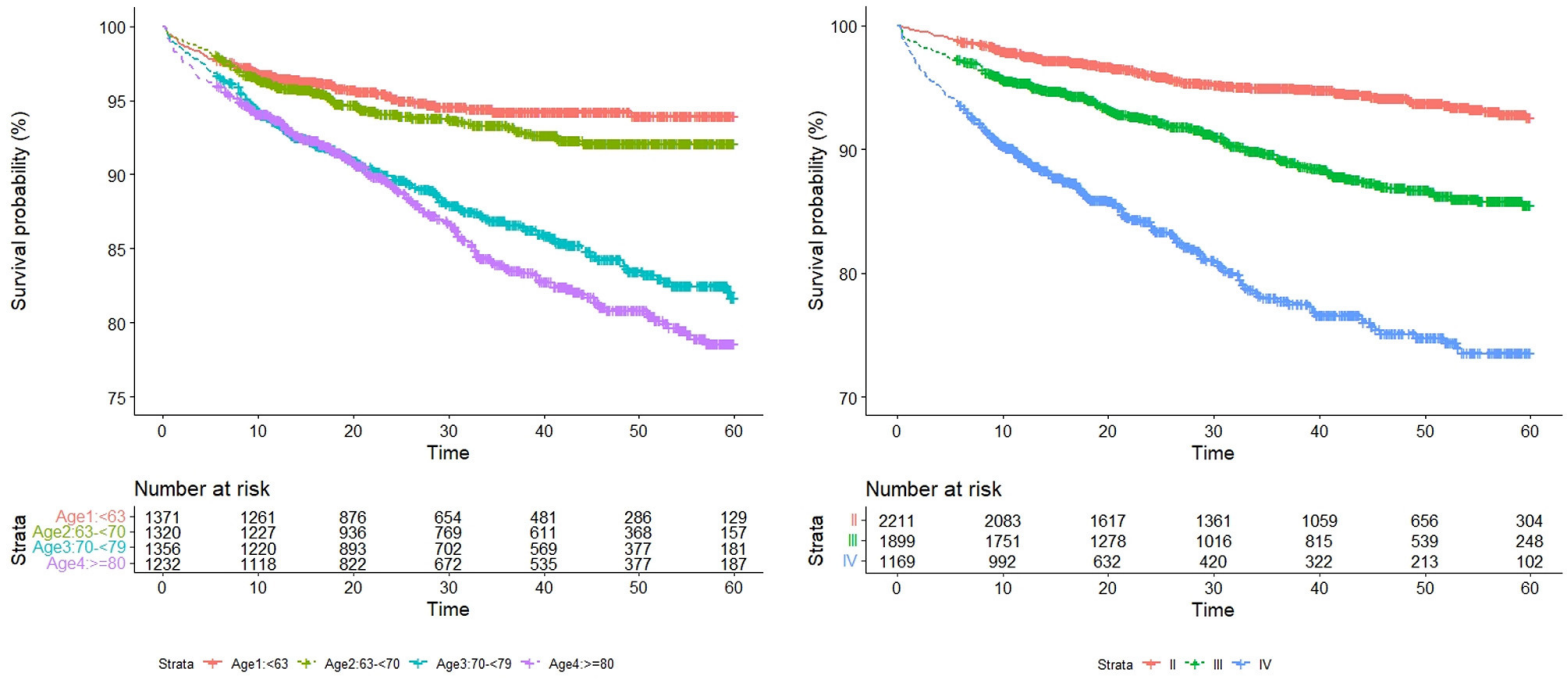
Cumulative survival probability of age and NYHA.

Univariate Cox analysis results are as follows (Table 1). In Figure 3, we show the survival curves of patients by age and NYHA subgroups.

**Table 1:** Univariate Cox regression of time to death.

**variables**	*****β_*b*_*****	***SE***	**Waldχ^2^**	***P***	**HR**	**HR95%CI**
Age (<63 as reference, *n* = 1,371)			90.291	<0.001		
Age (63 – <70, *n* = 1,320)	0.205	0.163	1.586	0.208	1.228	(0.892, 1.690)
Age (70 – <79, *n* = 1,356)	0.933	0.144	42.117	<0.001	2.541	(1.917, 3.368)
Age (≥80, *n* = 1232)	1.086	0.142	58.251	<0.001	2.962	(2.241, 3.914)
NYHA (II as reference,*n* = 2,211)			172.134	<0.001		
III (*n* = 1,899)	0.751	0.119	39.866	<0.001	2.119	(1.678, 2.675)
IV (*n* = 1,169)	1.507	0.117	165.613	<0.001	4.512	(3.587, 5.675)
**Comorbidity**						
PMI	0.391	0.088	19.782	<0.001	1.479	(1.245, 1.757)
Atrial fibrillation	0.470	0.092	26.181	<0.001	1.601	(1.337, 1.917)
VHD	0.565	0.127	19.701	<0.001	1.759	(1.371, 2.257)
Diabetes	0.322	0.091	12.418	<0.001	1.380	(1.154, 1.650)
Renal insufficiency	0.894	0.106	70.828	<0.001	2.444	(1.985, 3.01)
Cancer	0.662	0.108	37.346	<0.001	1.939	(1.568, 2.397)
**Medication use**						
Oral anticoagulants	−0.479	0.125	14.629	<0.001	0.619	(0.484, 0.792)
Statin	−0.682	0.104	42.763	<0.001	0.506	(0.412, 0.620)
β-blockers	−0.491	0.093	28.022	<0.001	0.612	(0.510, 0.734)
Aldosterone	0.595	0.098	37.001	<0.001	1.812	(1.496, 2.195)
Diuretic	0.956	0.099	93.978	<0.001	2.603	(2.145, 3.158)
Cardiac stimulant	0.855	0.099	74.637	<0.001	2.352	(1.937, 2.856)
**In hospital examination**						
Breaths per minute	0.295	0.101	8.569	0.003	1.343	(1.102, 1.635)
DBP (mmHg)	−0.444	0.089	25.190	<0.001	0.641	(0.539, 0.763)
BMI (Kg/m^2^)	−0.628	0.092	47.007	<0.001	0.534	(0.446, 0.639)
Heart rate per minute	0.477	0.091	27.285	<0.001	1.611	(1.347, 1.926)
WBC (10^12^/L)	0.256	0.089	8.215	0.004	1.291	(1.084, 1.538)
RBC (10^12^/L)	−0.438	0.090	23.774	<0.001	0.646	(0.541, 0.770)
RDW (%)	1.074	0.100	116.48	<0.001	2.928	(2.409, 3.559)
hemoglobin (g/L)	−0.524	0.091	33.435	<0.001	0.592	(0.496, 0.707)
ANC (10^10^/L)	0.546	0.091	36.098	<0.001	1.727	(1.445, 2.064)
NEUT (%)	0.888	0.096	86.172	<0.001	2.431	(2.015, 2.933)
ALT (U/L)	−0.199	0.088	5.082	0.024	0.820	(0.690, 0.974)
albumin (g/L)	−0.920	0.097	90.183	<0.001	0.398	(0.329, 0.482)
DBIL (μmol/L)	0.757	0.095	64.122	<0.001	2.133	(1.772, 2.567)
γGT (U/L)	0.518	0.090	33.090	<0.001	1.679	(1.407, 2.003)
Blood glucose (mmol/L)	0.312	0.09	12.138	<0.001	1.366	(1.146, 1.628)
TC (mmol/L)	−0.391	0.089	19.202	<0.001	0.676	(0.568, 0.806)
Triglyceride (mmol/L)	−0.762	0.093	66.951	<0.001	0.467	(0.389, 0.560)
LDL (μmol/L)	−0.382	0.089	18.331	<0.001	0.682	(0.573, 0.813)
BUN (mmol/L)	0.713	0.092	59.844	<0.001	2.040	(1.703, 2.445)
creatinine (mmol/L)	0.816	0.094	75.023	<0.001	2.262	(1.881, 2.721)
Uric acid (μmol/L)	0.634	0.091	48.406	<0.001	1.885	(1.577, 2.253)
Serum sodium (mmol/L)	−0.466	0.088	27.974	<0.001	0.628	(0.528, 0.746)
Serum chlorine (mmol/L)	−0.655	0.090	52.395	<0.001	0.519	(0.435, 0.620)
Cystatin C (mg/L)	0.894	0.096	86.666	<0.001	2.445	(2.026, 2.952)
FT3 (umol/L)	−1.205	0.097	153.433	<0.001	0.300	(0.248, 0.363)
FT4 (pmol/L)	1.208	0.103	137.403	<0.001	3.346	(2.734, 4.095)
NT-proBNP (ng/L)	1.437	0.107	179.584	<0.001	4.208	(3.411, 5.193)
Cardiac troponin (μg/L)	0.877	0.099	78.197	<0.001	2.405	(1.980, 2.921)
QRS (ms)	0.312	0.091	11.827	0.001	1.366	(1.143, 1.631)
QTC (ms)	0.519	0.091	32.804	<0.001	1.680	(1.407, 2.007)
LVEF (%)	−0.740	0.092	64.401	<0.001	0.477	(0.398, 0.572)

*NYHA, New York Heart Association; PMI, previous myocardial infarction, acute myocardial infarction occurred 6 months ag; VHD, valvular heart disease; renal insufficiency, previous symptoms of renal insufficiency were diagnosed by two attending physicians; oral anticoagulants, warfarin, aspirin, heparin, clopidogrel hydrogen sulfate tablet; DBP, diastolic blood pressure; WBC, white blood cells; RDW, red blood cell distribution width; ANC, absolute neutrophil count; NEUT, the neutrophils ratio; DBIL, direct bilirubin; TC, total cholesterol; LDL, low-density lipoprotein; BUN, blood urea nitrogen; FT3, free triiodothyronine; FT4, free thyroxine; LVEF, left ventricular ejection fraction (P <0.05, the difference was statistically significant)*.

### Feature Selection

**Figure 4:**
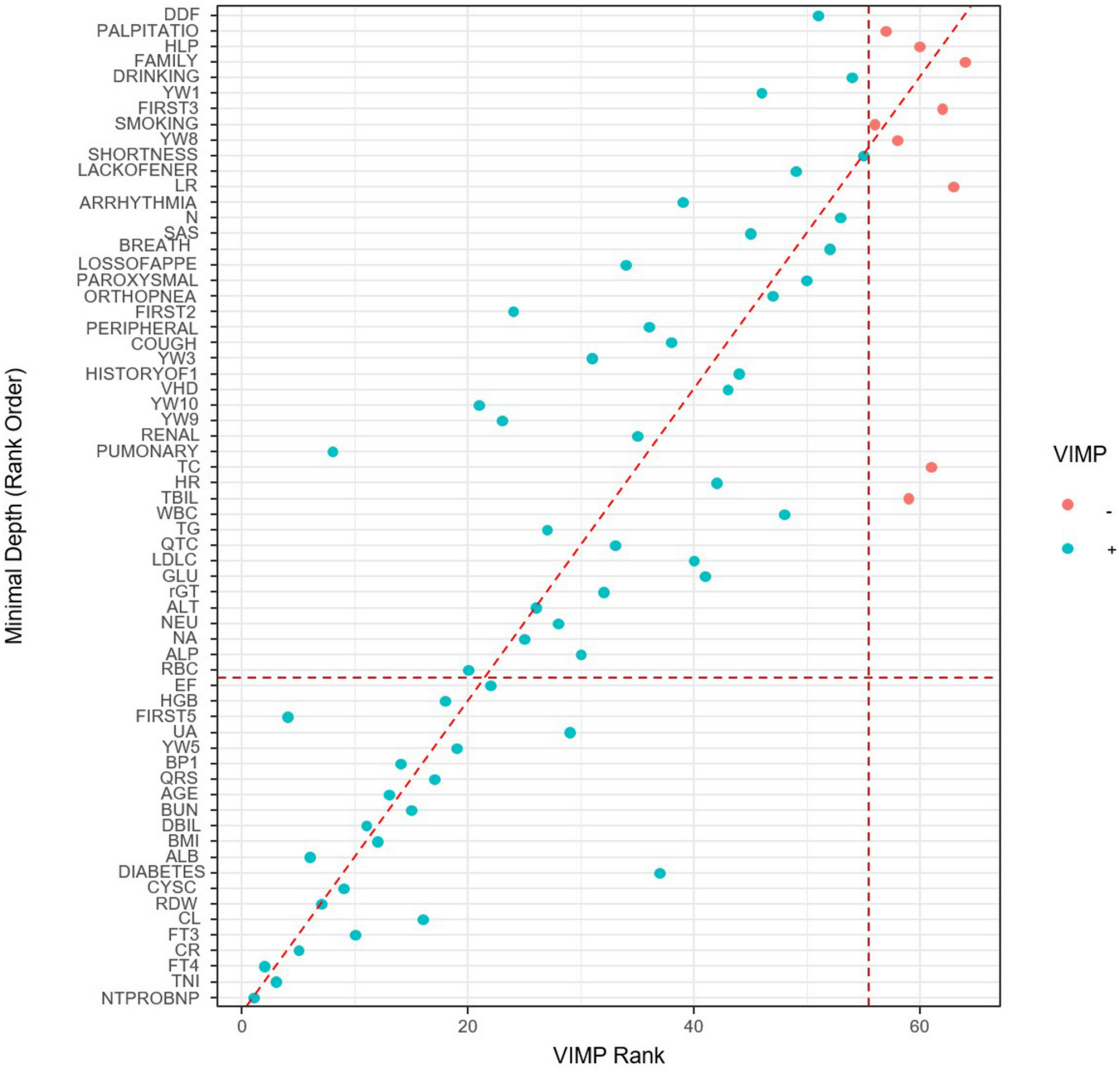
Variables selected by VIMP and minimal depth.

The RSF model was used to prioritize and explain the influencing factors using VIMP and Minimal Depth to select variables. The importance of the relationship between each attribute (predictor) to outcome were plotted with different colored dots, red for low-risk values and blue for high-risk values. Twenty-one Variables selected by both methods were selected for subsequent modeling (variables below the horizontal dotted line) (Figure 4, Table 2) (Details in Supplementary Figure 1).

**Table 2:** Results of selected variables in the final model.

**Variables**	*****β_*b*_*****	***SE***	**Wald**χ^2^****	***P***	**HR**	**HR95%CI**
Age (<63 as reference)			19.789	<0.001		
Age (63 – <70)	0.130	0.164	0.625	0.429	1.139	(0.825, 1.571)
Age (70 – <79)	0.567	0.146	15.025	<0.001	1.762	(1.323, 2.347)
Age (≥80)	0.369	0.149	6.104	0.013	1.446	(1.079, 1.937)
NYHA (II as reference)			14.331	0.001		
III	0.335	0.124	7.352	0.007	1.398	(1.097, 1.782)
IV	0.510	0.135	14.245	<0.001	1.665	(1.278, 2.170)
LVEF (%)	−0.288	0.095	9.270	0.002	0.749	(0.622, 0.902)
β-blockers	0.224	0.104	4.635	0.031	1.251	(1.020, 1.534)
Uric acid (μmol/L)	0.323	0.100	10.364	0.001	1.381	(1.135, 1.679)
ANC (10^10^/L)	0.016	0.005	12.947	<0.001	1.016	(1.007, 1.026)
DBP (mmHg)	−0.012	0.004	10.833	0.001	0.988	(0.981, 0.995)
QRS (ms)	0.002	0.001	6.334	0.012	1.002	(1.001, 1.004)
BUN (mmol/L)	0.308	0.106	8.487	0.004	1.361	(1.105, 1.673)
DBIL (μmol/L)	0.207	0.100	4.290	0.038	1.23	(1.011, 1.496)
BMI (Kg/m2)	−0.047	0.013	13.497	<0.001	0.954	(0.930, 0.978)
albumin (g/L)	−0.035	0.009	15.066	<0.001	0.966	(0.949, 0.983)
Diabetes	0.241	0.094	6.607	0.010	1.273	(1.059, 1.530)
Cystatin C (mg/L)	0.296	0.131	5.111	0.024	1.345	(1.040, 1.739)
FT3 (pmol/L)	−0.150	0.065	5.330	0.021	0.861	(0.758, 0.978)
FT4 (umol/L)	0.061	0.015	16.952	<0.001	1.063	(1.032, 1.094)
NT-proBNP(ng/L)	0.580	0.125	21.572	<0.001	1.786	(1.398, 2.282)
Cardiac troponin (μg/L)	0.289	0.099	8.486	0.004	1.335	(1.099, 1.622)
RDW (%)	0.370	0.106	12.26	<0.001	1.447	(1.177, 1.780)
Serum chlorine (mmol/L)	0.227	0.106	4.618	0.032	1.255	(1.020, 1.544)
Creatinine (μmol/L)	0.003	0.001	5.970	0.015	1.003	(1.001, 1.005)

*P < 0.05, the difference was statistically significant*.

### Interpretation of Predictive Features

**Figure 5:**
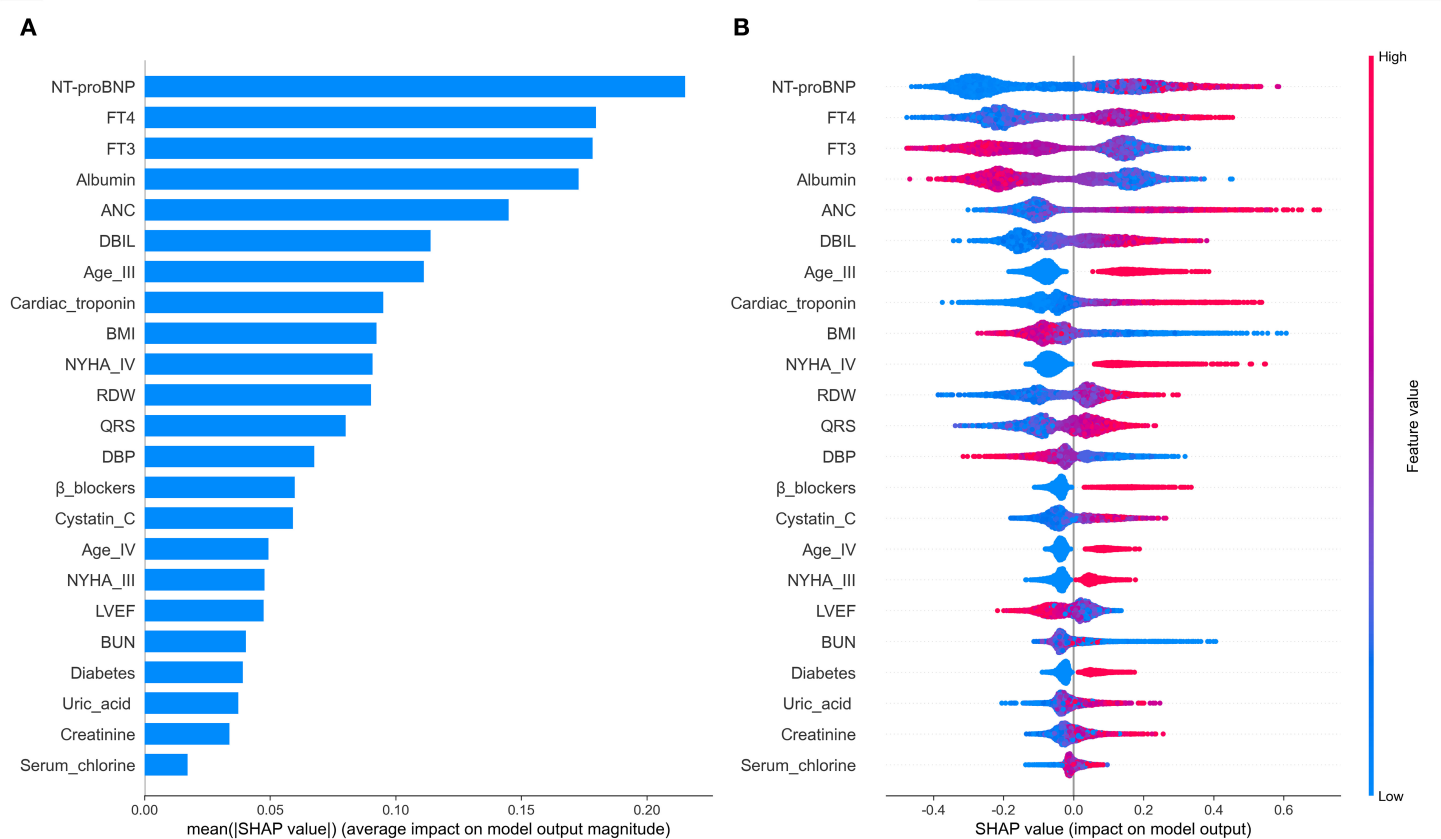
The model's interpretation. **(A)** The importance ranking of the variables according to the mean (|SHAP value|); **(B)** The importance ranking of the risk factors with stability and interpretation using the RSF model.

In order to explain the selected variables intuitively, we use SHAP (SHapley Additive exPlanations) (46) to illustrate how these variables affect the mortality rate in the model. Figure 5A shows the 21 risk factors assessed by the average absolute SHAP value. Figure 5B shows the details of the features in the model. The feature ranking (y-axis) indicates the importance of the predictive model. The SHAP value (x-axis) is a unified index that responds to the influence of a certain feature in the model. In each feature important row, use different colored dots to draw the attribution of all patients to the results, where the red dot represents the high-risk value, and the blue dot represents the low-risk value.

Older age, elevated NYHA Classification, a higher Uric acid, absolute neutrophil count, QRS, Blood urea nitrogen, direct bilirubin, Cystatin C, free thyroxine, NT-proBNP, Cardiac troponin, red blood cell distribution width, Serum chlorine, Creatinine; the presence of previous diabetes mellitus and noβ-blockers have increased the risk of CHF-related mortality. Furthermore, a lower blood pressure, BMI, albumin, left ventricular ejection fraction and free triiodothyronine were also associated with a higher predicted probability of CHF-related mortality.

Lasso Cox, RSF, and ELM Cox were then applied to construct the survival prediction models for CHF. In 2017, Voors (47) developed and validated a mortality risk model based on the clinical data of patients with heart failure with preserved ejection fraction from 11 European countries in the BIOSTAT-CHF and showed that advanced age, higher BUN and NT-proBNP, lower hemoglobin, and no β-blocker were the five variables with the strongest prediction effect on mortality, among which age, BUN, NT-proBNP, and β-blockers were consistent with the results of this paper.

### Model Prediction Performance Comparison

**Figure 6:**
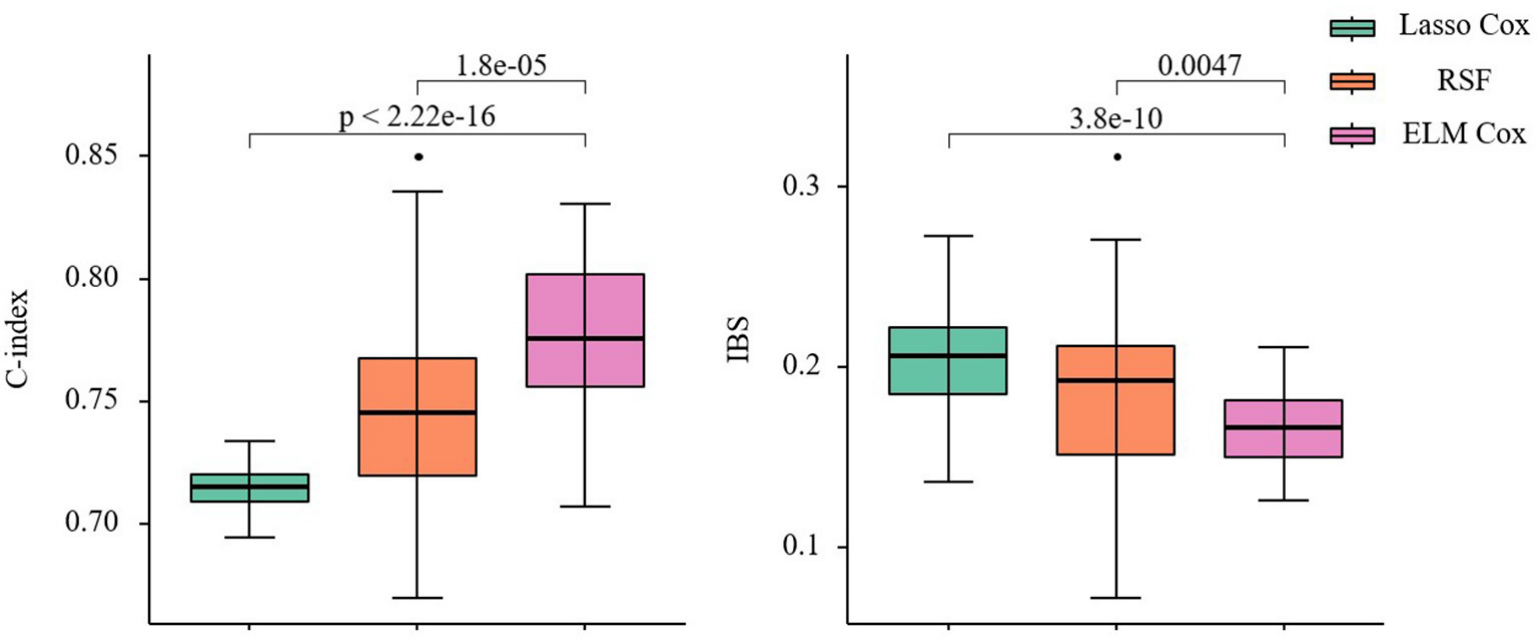
C-index and IBS of the three prediction models. Nonparametric Friedman test and Nemenyi *post hoc* test were used to make comparison with the ELM Cox group, *P* < 0.05 means statistically significant.

As shown in Figure 6, compared to the other two models, the ELM Cox model has the highest C-index 0.775(0.755, 0.802) and the lowest IBS 0.166(0.150, 0.182), showing the best overall performance. The results from the data application align with those from the simulation studies in this manuscript, and it can be concluded that the Cox proportional hazard model based on ELM could produce better predictions when applied to the survival analysis of patients with CHF.

## Discussion

Traditionally, the Cox proportional hazard regression algorithm is used to construct models for heart failure research, but its application conditions are subject to many restrictions (34).

In this study, the predictive performance of three survival analysis models, Lasso cox, RSF, and ELM Cox models, on a simulated dataset and an actual CHF dataset was compared. The prediction performance of the three models under three survival time data censoring ratios was compared, and the results showed that the prediction performance of the three models gradually decreases as the censoring ratio increases. However, the ELM Cox model performed the best with the highest stability. The simulation study laid the foundation for the study of actual CHF data and explored the possibility of constructing chronic disease survival analysis models on survival tie data with large censoring ratios.

In this paper, the Lasso Cox and RSF models consumed relatively longer training time on real data, especially when the RSF cross-validation is used to select the optimal parameters, each iteration taking 5–10 min. In addition to the short computational time, the evaluation metrics of the ELM Cox heart failure prediction model (C-index and IBS: 0.775, 0.166, respectively) were also the most ideal among the three models. Compared with the performance of the Lasso Cox and RSF models, the ELM Cox model showed stable performances on simulated and real data, which was still superior even with high censoring ratios.

The innovation of this study is that the classical parametric or semiparametric survival analysis model has serious limitations and cannot achieve good predictive effects in complex variables. For example, in the Cox risk proportional model, there are proportional hazards and log-linear assumptions. It is difficult to fully analyze the nonlinear relationship between the independent variable and the dependent variable. It is assumed that the risk ratio is constant over time (18). However, these basic assumptions are not easy to satisfy and difficult to verify in practice. In this study, a newer ELM Cox algorithm can be used to make up for the shortcomings of the traditional algorithm, and from the perspective of model construction, the algorithm is applied to the survival prediction of patients with chronic heart failure. It can improve the predictive ability of the survival model.

In this study, three survival prediction models, Lasso Cox, RSF, and ELM Cox models were constructed using electronic medical records of patients with CHF, with the following limitations: (1) This study analyzed survival censored higher proportion, 90.6%; thus, the C-index of the models was not very high; In the real-world high censored heart failure data research, there is no further comparison with established approaches that combine backpropagation-trained deep neural networks with Cox proportional hazards models and other integrated algorithms (29, 48), (2) The ELM Cox model is a black box when it comes to how the variables are used, a characteristic of all neural networks, and the intermediate links in building the model are not yet clear, (3) The data sources are only from Taiyuan city, Shanxi Province. Therefore, it is necessary to expand the sample sources in future studies, and (4) The models are constructed without external validation, which may be added in future studies.

## Conclusion

Overall, this study applies a newer survival analysis algorithm, the ELM Cox model, to build a survival prediction model for patients with CHF, which has a better and more stable prediction performance compared with the Lasso Cox and RSF models. The 21 clinical variables with a significant impact on the survival of heart failure patients are of great theoretical significance and application value in assessing the mortality risk of heart failure patients, assisting physicians to carry out targeted therapeutic measures for high-risk groups with poor prognosis, and preventing and mitigating the development of poor prognosis in CHF patients.

## Data Availability Statement

The raw data supporting the conclusions of this article will be made available by the authors, without undue reservation.

## Ethics Statement

The research program received medical and ethical approval from Shanxi Medical University (NO. 2018LL128). Written informed consent to participate in this study was provided by the participants or their legal guardian/next of kin.

## Author Contributions

HY conceived the study, designed the study protocol, analyzed and interpreted the data, and draft and write the manuscript. JT revised and reviewed the article. BM, KW, CZ, YL, and JY were responsible for collecting the data. HY and BM participated in the data analysis. QH and YZ came up with the original concept for the study, oversaw the data analysis, and revised the paper. All authors contributed to the article and approved the submitted version.

## Funding

This study was funded by the National Nature Science Foundation of China (Grant no. 81872714, 82173631) and the Shanxi Provincial Key Laboratory of Major Diseases Risk Assessment (Grant no. 201805D111006).

## Conflict of Interest

The authors declare that the research was conducted in the absence of any commercial or financial relationships that could be construed as a potential conflict of interest.

## Publisher's Note

All claims expressed in this article are solely those of the authors and do not necessarily represent those of their affiliated organizations, or those of the publisher, the editors and the reviewers. Any product that may be evaluated in this article, or claim that may be made by its manufacturer, is not guaranteed or endorsed by the publisher.

## References

[1] Alba C Agoritsas T Jankowski M Courvoisier D Walter SD Guyatt GH. Ross: risk prediction models for mortality in ambulatory patients with heart failure: a systematic review. Circ Heart Fail. (2013) 6:881–9. 10.1161/CIRCHEARTFAILURE.112.000043 23888045

[2] Jones NR Roalfe AK Adoki I Hobbs FR Taylor CJ. Survival of patients with chronic heart failure in the community: a systematic review and meta-analysis. Eur J Heart Fail. (2019) 21:1306–25. 10.1002/ejhf.1594 31523902 PMC6919428

[3] Mcmurray JJV Pfeffer MA. Heart failure. Lancet. (2005) 365:1877–89. 10.1016/S0140-6736(05)66621-4 15924986

[4] Zhou C Li A Hou A Zhang Z Zhang Z Dai P Wang F. Modeling methodology for early warning of chronic heart failure based on real medical big data. Expert Syst Appl. (2020) 151:113361. 10.1016/j.eswa.2020.113361

[5] Miller DD. Machine intelligence in cardiovascular medicine. Cardiol Rev. (2020) 28:53–64. 10.1097/CRD.0000000000000294 32022759

[6] Lyle M Wan SH Murphree D Bennett C Wiley BM Barsness G. Predictive value of the get with the guidelines heart failure risk score in unselected cardiac intensive care unit patients. J Am Heart Assoc. (2020) 9:e012439. 10.1161/JAHA.119.012439 31986993 PMC7033864

[7] Levy WC Mozaffarian D Linker DT Sutradhar SC Anker SD Cropp AB. The seattle heart failure model: prediction of survival in heart failure. Circulation. (2006) 113:1424–33. 10.1161/CIRCULATIONAHA.105.584102 16534009

[8] Bohra Worland T Hui S Terbah R Farrell A Robertson M. Prognostic significance of hepatic encephalopathy in patients with cirrhosis treated with current standards of care. World J Gastroenterol. (2020) 26:2221–31. 10.3748/wjg.v26.i18.2221 32476788 PMC7235207

[9] Taslimitehrani V Dong GZ Pereira NL Panahiazar M Pathak J. Developing EHR-driven heart failure risk prediction models using CPXR (Log) with the probabilistic loss function. J Biomed Inform. (2016) 60:260–69. 10.1016/j.jbi.2016.01.009 26844760 PMC4886658

[10] Eleuteri Tagliaferri R Milano L De Placido S De Laurentiis M. A novel neural network-based survival analysis model. Neural Netw. (2003) 16:855–64. 10.1016/S0893-6080(03)00098-4 12850044

[11] Hong N Wen A Stone DJ Tsuji S Kingsbury PR Rasmussen LV. Developing a FHIR-based EHR phenotyping framework: a case study for identification of patients with obesity and multiple comorbidities from discharge summaries. J Biomed Inform. (2019) 99:103310. 10.1016/j.jbi.2019.103310 31622801 PMC6990976

[12] Panahiazar M Taslimitehrani V Pereira NL Pathak J. Using EHRs for heart failure therapy recommendation using multidimensional patient similarity analytics. Stud Health Technol Inform. (2015) 210:369–73. 10.3233/978-1-61499-512-8-369 25991168 PMC4899831

[13] Mathur P Srivastava S Xu X Mehta JL. Artificial intelligence, machine learning, cardiovascular disease. Clin Med Insights Cardiol. (2020) 14:1179546820927404. 10.1177/1179546820927404 32952403 PMC7485162

[14] Wang Y Zhu K Li Y Lv Q Fu G Zhang W. A machine learning-based approach for the prediction of periprocedural myocardial infarction by using routine data. Cardiovasc Diagn Ther. (2020) 10:1313–24. 10.21037/cdt-20-551 33224755 PMC7666922

[15] Yin X Zhang F Guo H Peng C Zhang W Xiao J. A nomogram to predict the risk of hepatic encephalopathy after transjugular intrahepatic portosystemic shunt in cirrhotic patients. Sci Rep. (2020) 10:9381. 10.1038/s41598-020-65227-2 32523059 PMC7287049

[16] Attar R Wester A Koul S Eggert S Polcwiartek C Jernberg T. Higher risk of major adverse cardiac events after acute myocardial infarction in patients with schizophrenia. Open Heart. (2020) 7:e001286. 10.1136/openhrt-2020-001286 32994353 PMC7526274

[17] Koelling TM Joseph S Aaronson KD. Heart failure survival score continues to predict clinical outcomes in patients with heart failure receiving beta-blockers. J Heart Lung Transplant. (2004) 23:1414–22. 10.1016/j.healun.2003.10.002 15607672

[18] Weathers B. Comparision of Survival Curves Between Cox Proportional Hazards, Random Forests, and Conditional Inference Forests in Survival Analysis. Logan, UH: Utah State University (2017).

[19] Duggal B Subramanian J Duggal M Singh P Rajivlochan M Saunik S. Survival outcomes post percutaneous coronary intervention: why the hype about stent type? lessons from a healthcare system in India. PLoS ONE. (2018) 13:e0196830. 10.1371/journal.pone.0196830 29795604 PMC5967815

[20] Steele J Denaxas SC Shah AD Hemingway H Luscombe NM. Machine learning models in electronic health records can outperform conventional survival models for predicting patient mortality in coronary artery disease. PLoS ONE. (2018) 13:e0202344. 10.1371/journal.pone.0202344 30169498 PMC6118376

[21] Dietrich S Floegel A Troll M Kuhn T Rathmann W Peters A. Random survival forest in practice: a method for modelling complex metabolomics data in time to event analysis. Int J Epidemiol. (2016) 45:1406–20. 10.1093/ije/dyw145 27591264

[22] Miao F Cai Y-P Zhang Y-T Li C-Y. Is random survival forest an alternative to cox proportional model on predicting cardiovascular disease? In: 6th European Conference of the International Federation for Medical and Biological Engineering. Cham: Springer (2015).

[23] Wang H Li G. Extreme learning machine cox model for high-dimensional survival analysis. Stat Med. (2019) 38:2139–56. 10.1002/sim.8090 30632193 PMC6498851

[24] Ismaeel S Miri A Chourishi D. Using the extreme learning machine (ELM) technique for heart disease diagnosis. In: 2015 IEEE Canada International Humanitarian Technology Conference (IHTC2015). IEEE, Ottawa, ON, Canada (2015).

[25] Wang H Wang JX Zhou LF. A survival ensemble of extreme learning machine. Artif Intell. (2018) 48:1846–58. 10.1007/s10489-017-1063-4

[26] Ponikowski P Voors AA Anker SD Bueno H Cleland JGF Coats AJS. 2016 ESC guidelines for the diagnosis and treatment of acute and chronic heart failure. Eur J Heart Fail. (2016) 18:891–975. 10.1093/eurheartj/ehw128 27207191

[27] Yancy CW Jessup M Bozkurt B Butler J Casey DE Jr Colvin MM. 2017 ACC/AHA/HFSA focused update of the 2013 ACCF/AHA guideline for the management of heart failure: a report of the American College of Cardiology/American Heart Association task force on clinical practice guidelines and the Heart Failure Society of America. J Am Coll Cardiol. (2017) 70:776–803. 10.1016/j.cardfail.2017.04.014 28461007

[28] Ishwaran H Kogalur UB Chen X Minn AJ. Random survival forests for high-dimensional data. Stat Anal Data Min. (2011) 4:115–32. 10.1002/sam.1010330740388

[29] Wang H Zhou LF. SurvELM: an R package for high dimensional survival analysis with extreme learning machine. Knowl Based Syst. (2018) 160:28–33. 10.1016/j.knosys.2018.07.009

[30] Ishwaran H Kogalur UB Kogalur MUB. Package “randomForestSRC” (2020).

[31] Hastie T Qian J. Glmnet vignette (2014). Available online at: http://www.web.stanford.edu/~hastie/Papers/Glmnet_Vignette.pdf (accessed September 20, 2016).

[32] Bühlmann P. MissForest—non-parametric missing value imputation for mixed-type data. Bioinformatics. (2012) 28:112–8. 10.1093/bioinformatics/btr597 22039212

[33] Stekhoven DJ Stekhoven MDJ. Package “missForest” (2012).

[34] Tibshirani R. The lasso method for variable selection in the cox model. Stat Med. (1997) 16:385–95. 9044528 10.1002/(sici)1097-0258(19970228)16:4<385::aid-sim380>3.0.co;2-3

[35] Breiman L. Random forests. Mach Learn. (2001) 45:5–32. 10.1023/A:1010933404324

[36] Katzman JL Shaham U Cloninger A Bates J Jiang T Kluger Y. Deep survival: a deep cox proportional hazards network. stat. arXiv:1606.00931. (2016) 1050:1–10.

[37] Huang GB Zhu QY Siew CK. Extreme learning machine: a new learning scheme of feedforward neural networks. In: IEEE International Joint Conference on Neural Networks. Budapest (2005).

[38] Park J Sandberg I. Universal approximation using radial-basis-function networks. Neural Comput. (2014) 3:246–57. 10.1162/neco.1991.3.2.246 31167308

[39] Leshno M Ya.Lin V Pinkus A Schocken S. Multilayer feedforward networks with a nonpolynomial activation function can approximate any function. Neural Netw. (1993) 6:861–7. 10.1016/S0893-6080(05)80131-5

[40] Huang G-B Zhu Q-Y Siew C-K. Extreme learning machine: theory and applications. Neurocomputing. (2006) 70:489–501. 10.1016/j.neucom.2005.12.126

[41] Kawaguchi ES Suchard MA Liu Z Li G. Scalable sparse cox's regression for large-scale survival data *via* broken adaptive ridge. arXiv e-prints arXiv:1712.00561 (2017).

[42] Harrell FE. Regression modeling strategies: with applications to linear models, logistic and ordinal regression, survival analysis. New York, NY: Springer (2015).

[43] Chen C Liaw A Breiman L. Using random forest to learn imbalanced data. Berkeley, CA: University of California (2004). p. 24.

[44] Ghosh G Jesudian AB. Small intestinal bacterial overgrowth in patients with cirrhosis. J Clin Exp Hepatol. (2019) 9:257–67. 10.1016/j.jceh.2018.08.006 31024208 PMC6477138

[45] Brilleman SL Wolfe R Moreno-Betancur M Crowther MJ. Simulating survival data using the simsurv R Package. J Stat Softw. (2021) 97:1–27. 10.18637/jss.v097.i03

[46] Lundberg SM Erion G Chen H DeGrave A Prutkin JM Nair B. From local explanations to global understanding with explainable AI for trees. Nat Mach Intell. (2020) 2:56–67. 10.1038/s42256-019-0138-9 32607472 PMC7326367

[47] Voors A Ouwerkerk W Zannad F van Veldhuisen DJ Samani NJ Ponikowski P. Development and validation of multivariable models to predict mortality and hospitalization in patients with heart failure. Eur J Heart Fail. (2017) 19:627–34. 10.1002/ejhf.785 28247565

[48] Kvamme H Borgan Ø Scheel I. Time-to-Event Prediction With Neural Networks and Cox Regression. arXiv [Preprint] arXiv:1907.00825 (2019).

