# Supplementary material for: Application of Extreme Learning Machine in the Survival Analysis of Chronic Heart Failure Patients With High Percentage of Censored Survival Time
Source: Front Cardiovasc Med. 2021 Oct 29;8:726516. doi: 10.3389/fcvm.2021.726516 (PMC8586069; doi:10.3389/fcvm.2021.726516)
Supplement: Supplementary file 1 [file Data_Sheet_1.docx]

**Application of Extreme Learning Machine in the Survival Analysis of Chronic Heart Failure Patients with High Percentage of Censored Survival Time**

**Supplementary Material**

**1. Candidate Variables**

The patient information was collected according to the case report form of chronic heart failure (CHF-CRF) developed by our research group according to the case record content and HF guidelines. CHF-CRF included the patient’s demographic(e.g., sex, smoking, drinking), vital signs (e.g., height, weight, temperature, blood oxygen saturation), heart failure causes (e.g., coronary heart disease, myocardial infarction, angina pectoris, obesity), heart failure complications (e.g., diabetes, arrhythmia, renal insufficiency, sleep apnea syndrome), symptoms and signs (e.g., palpitations, chest suppress, difficulty breathing), laboratory examination (e.g., WBC, RBC, hemoglobin, NT-proBNP), auxiliary examination, (e.g., electrocardiogram, dynamic electrocardiogram), and other examinations within 24 hours after admission and treatments (electrocardiogram monitoring and blood pressure monitoring), drug therapy (e.g., diuretics, angiotensin-converting enzyme inhibitors, angiotensin receptor blockers, beta-blockers), and oxygen therapy.

According to the CHF-CRF, 276 variables that conform to this research have been preliminarily determined. We eliminated variables with missing information ≥30% and merged some variables (for example, based on the principles and effects of drug therapy, all 89 drugs were classified as antiplatelet, anticoagulant, statin, nitrate, beta receptor Blockers, ACEI, ARB, aldosterone, diuretics, cardiotonic, sedative hypnosis and other 11 categories). Then, random forest VIMP (variable Importance) and minimal depth methods are used to select variables, and finally, 21 variables are selected to build the model.

**2. Description of baseline features**

The categorical variables in the medical records were computed by chi-square test, represented by n (%); The continuous variables in the medical records were computed by the rank-based non-parametric test, represented by Md(P_25_, P_75_). P-value≤0.05 was considered statistically significant. The initially variables were shown in Supplementary Table 1.

Supplementary Table 1. Results of baseline data characteristics

| Variable | Outcome | | *Z/*$\chi^{2}$ | *P* |
| --- | --- | --- | --- | --- |
|  | Alive (N=4762) | Death (N=517) |  |  |
| Male | 3078(64.6) | 326(63.1) | 0.509 | 0.476 |
| Age | 69.39±11.60 | 74.53±11.35 | 9.758 | <0.001 |
| NYHA |  |  | 158.985 | <0.001 |
| II | 1955(42.3) | 93(18.7) |  |  |
| III | 1707(37.0) | 192(38.6) |  |  |
| IV | 956(20.7) | 213(42.8) |  |  |
| Smoking | 1396(29.3) | 126(24.4) | 6.206 | 0.045 |
| Drinking | 953(20.0) | 80(15.5) | 7.371 | 0.025 |
| Family history | 874(18.4) | 71(13.7) | 6.775 | 0.009 |
| Surgery history | 480(10.1) | 72(13.9) | 7.370 | 0.007 |
| PMI | 1874(39.4) | 259(50.1) | 22.355 | <0.001 |
| Atrial fibrillation | 1247(26.2) | 184(35.6) | 20.87 | <0.001 |
| CAD | 4280(89.9) | 472(91.3) | 1.043 | 0.307 |
| Hypertension | 2882(60.5) | 313(60.5) | 0.001 | 0.993 |
| VHD | 441(9.3) | 72(13.9) | 11.572 | 0.001 |
| HLP | 1100(23.1) | 72(13.9) | 22.72 | <0.001 |
| Diabetes | 1386(28.7) | 189(36.6) | 13.748 | <0.001 |
| CNS diseases | 1010(21.2) | 129(25.0) | 3.859 | 0.049 |
| Lung disease | 670(14.1) | 162(31.3) | 104.705 | <0.001 |
| Renal insufficiency | 483(10.1) | 114(22.1) | 65.927 | <0.001 |
| Cancer | 50(1.0) | 14(2.7) | 10.704 | 0.001 |
| Sleep disorder | 608(12.8) | 108(20.9) | 26.242 | <0.001 |
| Palpitation | 1221(25.6) | 105(20.3) | 7.047 | 0.008 |
| Chest tightness | 3795(79.7) | 420(81.2) | 0.691 | 0.406 |
| Shortness of breath | 3196(67.1) | 408(78.9) | 29.989 | <0.001 |
| Dizziness | 582(12.2) | 55(10.6) | 1.102 | 0.294 |
| Syncope | 90(1.9) | 12(2.3) | 0.457 | 0.499 |
| Orthopnea | 586(12.3) | 117(22.6) | 43.068 | <0.001 |
| Paroxysmal dyspnea | 683(14.3) | 110(21.3) | 17.565 | <0.001 |
| Reduced exercise endurance | 1331(28.0) | 145(28.0) | 0.002 | 0.963 |
| Fatigue | 691(14.5) | 62(12.0) | 2.419 | 0.12 |
| Cough | 840(17.6) | 163(31.5) | 58.452 | <0.001 |
| Loss of appetite | 985(20.7) | 184(35.6) | 60.098 | <0.001 |
| Lack of energy | 865(18.2) | 159(30.8) | 47.279 | <0.001 |
| Weight loss | 94(2.0) | 14(2.7) | 1.254 | 0.263 |
| Ankle edema | 935(19.6) | 171(33.1) | 50.872 | <0.001 |
| Peripheral edema | 833(17.5) | 152(29.4) | 43.570 | <0.001 |
| Rale | 1308(27.5) | 233(45.1) | 69.892 | <0.001 |
| DDF | 2925(61.4) | 281(54.4) | 9.780 | 0.002 |
| Antiplatelet | 4222(88.7) | 442(85.5) | 4.545 | 0.033 |
| Oral anticoagulants | 501(10.5) | 48(9.3) | 0.765 | 0.382 |
| Statin | 4118(86.5) | 397(76.8) | 35.356 | <0.001 |
| Nitrate | 3572(75.0) | 376(72.7) | 1.289 | 0.256 |
| β-blockers | 3636(76.4) | 340(65.8) | 28.136 | <0.001 |
| ACEI | 1323(27.8) | 130(25.1) | 1.626 | 0.202 |
| ARB | 1181(24.8) | 117(22.6) | 1.184 | 0.276 |
| Aldosterone | 2814(59.1) | 371(71.8) | 31.269 | <0.001 |
| Diuretic | 2452(51.5) | 375(72.5) | 83.022 | <0.001 |
| Cardiac stimulant | 630(13.2) | 140(27.1) | 71.801 | <0.001 |
| Sedative hypnotics | 490(10.3) | 59(11.4) | 0.630 | 0.427 |
| After PCI | 899(18.9) | 65(12.6) | 12.425 | <0.001 |
| After CABG | 381(8.9) | 27(5.2) | 5.048 | 0.025 |
| Temp(℃） | 36.4(36.2,36.5) | 36.35(36.2,36.5) | 1.618 | 0.106 |
| Breaths per minute | 19(18,20) | 20(19,20) | 3.761 | <0.001 |
| SBP(mmHg) | 130(116,141) | 132(118,150) | 0.458 | 0.647 |
| DBP (mmHg) | 80(70,88) | 76(70,88) | -5.707 | <0.001 |
| BMI(Kg/m2) | 24.88±3.61 | 23.47±3.82 | -7.901 | <0.001 |
| Heart rate per minute | 73(64,85) | 78(64,90) | 6.495 | <0.001 |
| WBC (10^12/L) | 6.8(5.6,8.5) | 6.75(5.7,8.1) | 3.118 | 0.002 |
| RBC (10^12/L) | 4.44(4.03,4.86) | 4.18(3.9,4.52) | -6.885 | <0.001 |
| RDW (%) | 13.9(13.3,14.6) | 14.4(13.7,15.4) | 9.982 | <0.001 |
| hemoglobin (g/L) | 137(125,149) | 132(120,139) | -7.191 | <0.001 |
| PLT(10^9/L) | 183(151,229) | 172(147,201) | -2.265 | 0.023 |
| ANC (10^9/L) | 4.49(3.41,5.655) | 4.79(3.75,5.61) | 6.402 | <0.001 |
| NEUT (%) | 64.9(58.35,72.2) | 69.2(63.3,75) | 10.018 | <0.001 |
| ALT(U/L) | 18(13,26.65) | 17(12,31) | -4.664 | <0.001 |
| AST(U/L) | 22(17,29) | 20.5(18,29) | 0.517 | 0.605 |
| albumin (g/L) | 43.6(40,47) | 41(40,44) | -10.919 | <0.001 |
| TBIL (μmol/L) | 13.8(10.8,19.6) | 14.4(10.7,19.6) | 1.623 | 0.104 |
| DBIL (μmol/L) | 3.4(2.4,5.4) | 5.4(3.1,6.8) | 8.934 | <0.001 |
| IBIL (μmol/L) | 10.6(7.6,15) | 10.45(7.2,13.8) | -1.784 | 0.074 |
| ALP(U/L) | 74(62,90) | 74.5(57,90) | 1.447 | 0.148 |
| γGT(U/L) | 26(18.75,44) | 40(17,56) | 4.180 | <0.001 |
| TBA(μmol/L) | 4(2,6) | 3.5(2,6) | -0.540 | 0.589 |
| Blood glucose (mmol/L) | 4.9(4.3,6.1) | 4.07(3.48,4.99) | 2.866 | 0.004 |
| TC (mmol/L) | 4.07(3.48,4.99) | 3.75(3.35,4.52) | -3.673 | <0.001 |
| Triglyceride (mmol/L) | 1.35(0.98,1.79) | 1.095(0.83,1.65) | -6.756 | <0.001 |
| HDL(mmol/L) | 1.00(0.86,1.17) | 0.98(0.88,1.19) | 1.247 | 0.212 |
| LDL (mmol/L) | 2.43(1.97,3.15) | 2.35(1.91,2.90) | -4.468 | <0.001 |
| LP(α)(mg/L) | 144.1(82.3,278.7) | 139.55(91.3,244.3) | 1.580 | 0.114 |
| BUN (mmol/L) | 6.20(4.93,7.70) | 6.45(5.10,9.76) | 7.866 | <0.001 |
| creatinine (μmol/L) | 77(64,94) | 89.3(71.3,107.0) | 9.897 | <0.001 |
| Uric acid (μmol/L) | 374(299,450) | 398(330,519) | 6.774 | <0.001 |
| Serum potassium(mmol/L) | 4.00(3.72,4.26) | 4.13(3.76,4.40) | 3.838 | <0.001 |
| Serum sodium (mmol/L) | 140.5(138.5,142) | 140(138,142) | -4.394 | <0.001 |
| Serum chlorine (mmol/L) | 104.2(101.1,107) | 104(100.7,107) | -8.429 | <0.001 |
| Cystatin C (mg/L) | 1.1(0.9,1.4) | 1.125(0.95,1.5) | 9.07 | <0.001 |
| FT3 (pmol/L) | 4.38(3.925,4.845) | 4.06(3.51,4.5) | -7.771 | <0.001 |
| FT4 (pmol/L) | 12.19(10.74,14.09) | 12.52(10.66,15.59) | 7.491 | <0.001 |
| TSH(mlU/L) | 2.06(1.33,3.395) | 2(1.29,4.06) | 0.035 | 0.972 |
| NT-proBNP(ng/L) | 1079(311,3346) | 3074.5(1297,6595) | 11.521 | <0.001 |
| Cardiac troponin (μg/L) | 0.01(0.01,0.04) | 0.025(0.01,0.06) | 10.353 | <0.001 |
| USG(%) | 1.015(1.010,1.025) | 1.015(1.015,1.020) | -2.848 | 0.004 |
| QRS (ms) | 96(88,110) | 104(90,120) | 4.962 | <0.001 |
| QTC (ms) | 444(416,474) | 456(424,473) | 5.399 | <0.001 |
| LVEF(%) | 49(38,59) | 41(38,56) | -7.199 | <0.001 |

**NOTES:** CAD: Coronary artery disease; HLP: Hyperlipidemia; CNS: Central nervous system; DDF: Decreased diastolic function; ACEI: Angiotensin converting enzyme inhibitor; ARB: AngiotensinⅡ receptor blocker; PCI: Percutaneous coronary interventions; CABG: Coronary artery bypass grafting; SBP: Systolic blood pressure; PLT: Platelet; AST: Aspartate aminotransferase; TBIL: Serum total bilirubin; IBIL: Serum indirect bilirubin; ALP: Alkaline phosphatase; TBA: Serum total bile acid; HDL: High density lipoprotein; TSH: Thyroid stimulating hormone; USG: Urine specific gravity; NYHA: New York Heart Association; PMI: previous myocardial infarction, acute myocardial infarction occurred six months ag; VHD, valvular heart disease; renal insufficiency: previous symptoms of renal insufficiency were diagnosed by 2 attending physicians; oral anticoagulants: Warfarin, aspirin, heparin, Clopidogrel Hydrogen Sulfate Tablet; DBP: diastolic blood pressure; WBC: white blood cells; RDW: red blood cell distribution width; ANC: absolute neutrophil count; NEUT: The neutrophils ratio; DBIL: direct bilirubin; TC: Total cholesterol; LDL: Low-Density Lipoprotein; BUN: Blood urea nitrogen; FT3: free triiodothyronine; FT4: free thyroxine; LVEF: Left ventricular ejection fraction.

**3. Feature Selection**

VIMP (variable importance) method and Minimal Depth method are commonly used variable selection methods in random survival forest models. A variable VIMP value less than 0 indicates that the variable reduces the accuracy of prediction, and when the value of VIMP is greater than 0, it indicates that the variable improves the accuracy of prediction. The minimum depth law gives the importance of each variable to the final event by calculating the minimum depth when it runs to the final node. Supplementary Figure 1 is the variables filtered by the two methods respectively.


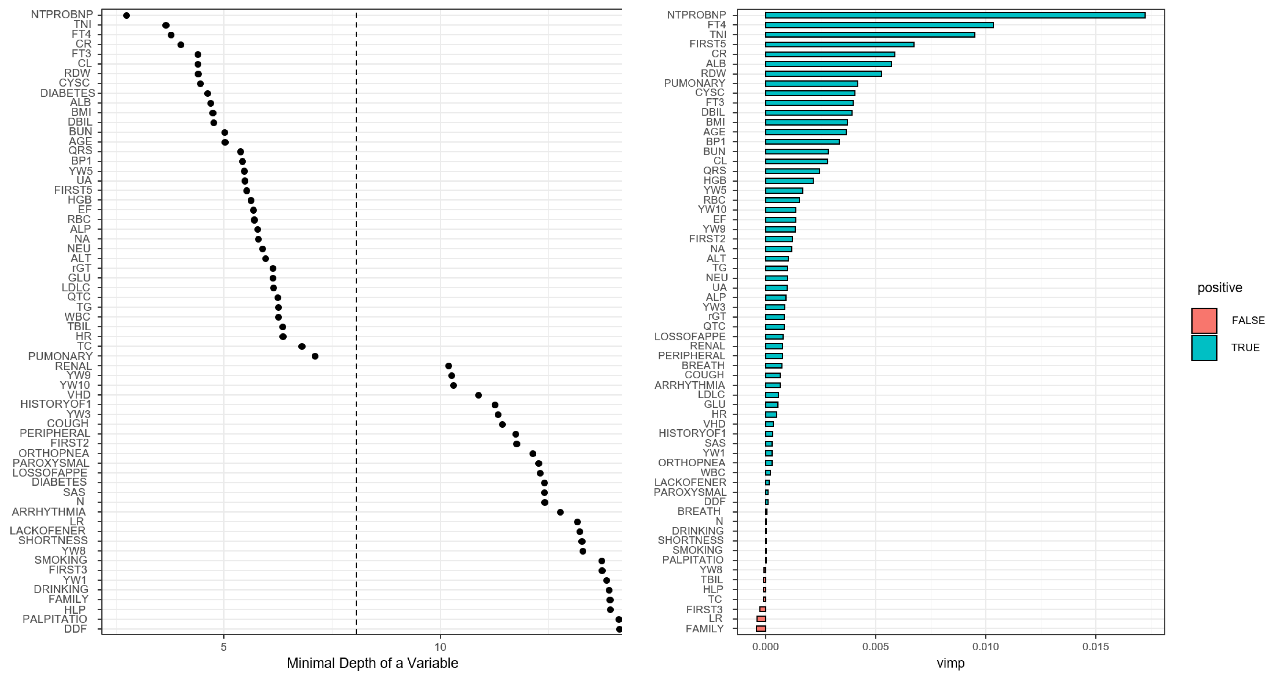


Supplementary Figure 1. VIMP and Minimal depth by variable importance for the variable random survival forests analysis.

**4. R package list**

Supplementary Table 2. R Packages function description

| R package | Version | Function | Description |
| --- | --- | --- | --- |
| *SurvELM* | 0.9.0 | ELMCox() | A Regularized Cox Extreme Learning Machine Model |
| *glmnet* | 4.1-2 | glmnet() | Fit a GLM with lasso |
| *randomForestSRC* | 2.12.0 | rfsrc() | Fast Unified Random Forests for Survival |
| *ggRandomForests* | 2.0.1 | var.select() | Select variable and visually exploring random forests |
| *SurvMetrics* | 0.3.5 | Cindex()\IBS() | Predictive Evaluation Metrics in Survival Analysis |
| *SimSurv* | 1.0.0 | simsurv() | Simulate Survival Data |
| *missForest* | 1.4 | missForest() | Nonparametric Missing Value Imputation using Random Forest |

**5. Feature missing ratio**

Supplementary Table 3. Variable missing proportional description

| Variable | Count | Percentage (%) |
| --- | --- | --- |
| *BNPN1* | *5209* | *96.91* |
| *EF1* | *5066* | *94.25* |
| *MYO* | *4720* | *87.81* |
| *HCYUMOLL* | *4418* | *82.2* |
| *MG* | *3971* | *73.88* |
| *CA* | *3582* | *66.64* |
| *CKMB* | *3120* | *58.05* |
| *ASTL* | *2489* | *46.31* |
| *TSH* | *2458* | *45.73* |
| *KK* | *2445* | *45.49* |
| *OCUPATION* | *2427* | *45.15* |
| *MCAD* | *1969* | *36.63* |
| *TNI* | *1940* | *36.09* |
| Cystatin C (mg/L) | 1424 | 26.49 |
| IBIL (μmol/L) | 1132 | 21.06 |
| TBIL (μmol/L) | 1132 | 21.06 |
| NT-proBNP(ng/L) | 1122 | 20.87 |
| FT3 (pmol/L) | 977 | 18.18 |
| FT4 (pmol/L) | 911 | 16.95 |
| Surgery history | 847 | 15.76 |
| PMI | 705 | 13.12 |
| Atrial fibrillation | 648 | 12.06 |
| CAD | 613 | 11.4 |
| Hypertension | 583 | 10.85 |
| VHD | 568 | 10.57 |
| HLP | 491 | 9.13 |
| Diabetes | 466 | 8.67 |
| CNS diseases | 457 | 8.5 |
| Lung disease | 457 | 8.5 |
| Renal insufficiency | 451 | 8.39 |
| Cancer | 418 | 7.78 |
| Sleep disorder | 396 | 7.37 |
| Palpitation | 372 | 6.92 |
| Chest tightness | 364 | 6.77 |
| Shortness of breath | 363 | 6.75 |
| Dizziness | 357 | 6.64 |
| Syncope | 282 | 5.25 |
| Orthopnea | 281 | 5.23 |
| Paroxysmal dyspnea | 273 | 5.08 |
| Reduced exercise endurance | 273 | 5.08 |
| Fatigue | 259 | 4.82 |
| Cough | 253 | 4.71 |
| Loss of appetite | 244 | 4.54 |
| Lack of energy | 227 | 4.22 |
| Weight loss | 224 | 4.17 |
| Ankle edema | 199 | 3.7 |
| Peripheral edema | 105 | 1.95 |
| Rale | 101 | 1.88 |
| DDF | 76 | 1.41 |
| Antiplatelet | 76 | 1.41 |
| Oral anticoagulants | 46 | 0.86 |
| Statin | 35 | 0.65 |
| Nitrate | 23 | 0.43 |
| β-blockers | 23 | 0.43 |
| ACEI | 17 | 0.32 |
| ARB | 14 | 0.26 |
| Aldosterone | 12 | 0.22 |
| Diuretic | 11 | 0.2 |
| Cardiac stimulant | 9 | 0.17 |
| Sedative hypnotics | 0 | 0 |
| After PCI | 0 | 0 |
| After CABG | 0 | 0 |
| Temp(℃） | 0 | 0 |
| Breaths per minute | 0 | 0 |
| SBP(mmHg) | 0 | 0 |
| DBP (mmHg) | 0 | 0 |
| BMI(Kg/m2) | 0 | 0 |
| Heart rate per minute | 0 | 0 |
| WBC (10^12/L) | 0 | 0 |
| RBC (10^12/L) | 0 | 0 |
| RDW (%) | 0 | 0 |
| hemoglobin (g/L) | 0 | 0 |
| PLT(10^9/L) | 0 | 0 |
| ANC (10^9/L) | 0 | 0 |
| NEUT (%) | 0 | 0 |
| ALT(U/L) | 0 | 0 |
| AST(U/L) | 0 | 0 |
| albumin (g/L) | 0 | 0 |
| NYHA | 0 | 0 |
| DBIL (μmol/L) | 0 | 0 |
| AGE | 0 | 0 |
| ALP(U/L) | 0 | 0 |
| γGT(U/L) | 0 | 0 |
| TBA(μmol/L) | 0 | 0 |
| Blood glucose (mmol/L) | 0 | 0 |
| TC (mmol/L) | 0 | 0 |
| Triglyceride (mmol/L) | 0 | 0 |
| HDL(mmol/L) | 0 | 0 |
| LDL (mmol/L) | 0 | 0 |
| LP(α)(mg/L) | 0 | 0 |
| BUN (mmol/L) | 0 | 0 |
| Creatinine (μmol/L) | 0 | 0 |
| Uric acid (μmol/L) | 0 | 0 |
| Serum potassium(mmol/L) | 0 | 0 |
| Serum sodium (mmol/L) | 0 | 0 |
| Serum chlorine (mmol/L) | 0 | 0 |
| GENDER | 0 | 0 |
| Drinking | 0 | 0 |
| Family history | 0 | 0 |
| TSH(mlU/L) | 0 | 0 |
| Smoking | 0 | 0 |
| Cardiac troponin (μg/L) | 0 | 0 |
| USG(%) | 0 | 0 |
| QRS (ms) | 0 | 0 |
| QTC (ms) | 0 | 0 |
| LVEF(%) | 0 | 0 |

*****The variables percentage (%)>30% were deleted.
